# A Case Report on Spinal Neurostimulator Treatment for Painful Postsurgical Neuropathy of the Genitofemoral Nerve

**DOI:** 10.7759/cureus.42345

**Published:** 2023-07-23

**Authors:** Juan J Medina-Pérez, Andrés Vega-Rosas, Luis Rodríguez-Méndez, Silvia G Coubert-Pelayo

**Affiliations:** 1 Pain Management Center, Hospital Ángeles Mocel, Mexico City, MEX; 2 Pain Clinic, Hospital Escandón, Mexico City, MEX

**Keywords:** inguinal hernioplasty, intractable pain, genitofemoral nerve, neuropathy, spinal neurostimulator

## Abstract

Painful postsurgical neuropathy is an adverse event inherent to a wide variety of surgical treatments, so its diagnosis and specialized treatment are essential to maintaining the quality of life of the people who suffer from it. We present the case of a 31-year-old male with neuropathy of the genitofemoral nerve diagnosed by electromyography, resulting in intractable left testicular and thigh pain associated with a recent history of ipsilateral inguinal hernioplasty. After assessment by pain medicine and motor and sensory tests, a neurostimulator was placed in T8-T9 with action at the L1-L2 level, as well as a simultaneous electrode in S3, generating optimal pain relief and recovery of functionality.

## Introduction

Painful postsurgical neuropathies are an infrequent but potentially devastating complication of a large number of surgical procedures and may be due to direct injury to a peripheral nerve or complex inflammatory processes. The perioperative causes, including anesthesia, traction, compression, and transection, are mechanisms where nerve damage can result in neuropathic pain that can evolve and become chronic, often being refractory to most conservative treatments and promoting excessive and ineffective consumption of painkillers. Considering the opioid epidemic, it is essential that all patients who present with postsurgical pain for more than three months be referred for a specialized evaluation by pain medicine [1]. In observational studies performed at the Mayo Clinic, postoperative nerve injury in orthopedic surgeries occurred in 2.2% of cases in the shoulder, 0.79% in the knee, and 0.72% after total hip arthroplasty, in such a way that even in the face of the most rigorous standards of clinical care, the risk of presenting this type of complications is present [2]. The use of neurostimulators (such as the implantation of a spinal neurostimulator and other types of electrodes in peripheral nerves) has been increasingly used to manage acute and chronic pain, especially in cases where conservative management has been shown to have low effectiveness. Peripheral nerve stimulation has demonstrated evidence for the treatment of peripheral neuropathic pain and postsurgical pain [3].

Neurostimulation modalities arose as a response to modulate the pain impulses provoked in the periphery, which are carried by C fibers and A-delta fibers, interrupting them by stimulating larger A-beta fibers at the common nerve synapse location in the substantia gelatinosa of the dorsal horn [4]. In this way, minimally invasive procedures with great selectivity can be offered by spinal cord stimulation, which has shown significant progress in the management of intractable pain through improvements in hardware and software, promoting greater effectiveness and reducing complications [5]. However, to maintain specificity in the approach, it is crucial to objectively document the nerve damage being considered. Electrophysiology studies such as electromyography and nerve conduction studies have proven useful for specifically testing nerve damage [6], especially in cases of painful neuropathies affecting the perineal area [7]. Considering the rapid progress in the development of neuromodulation devices, the Neurostimulation Appropriateness Consensus Committee has established recommendations for surgical planning, device placement, and postoperative care in the implementation of this type of treatment in the spinal cord [8], cervical [9], and dorsal root ganglion [10] stimulation for persistent outcome optimization.

## Case presentation

A previously healthy 31-year-old man arrived at the emergency room with a seven-day history of left scrotal and upper ipsilateral thigh pain, arising rapidly and progressively after the surgical approach of a right inguinal hernia. He experienced continuous pain that limited the optimal performance of the activities of daily life, significantly affected his quality of sleep, and diminished the enjoyment of his sexuality due to the exacerbation of pain.

Previously, the patient with no significant medical history attended a urological evaluation for experiencing intermittent and oppressive pain in the left testicle for five months; a bilateral direct inguinal hernia was diagnosed through physical examination and ultrasound. An uneventful bilateral laparoscopic hernioplasty was performed, and he was discharged home after the procedure. After a partial improvement in pain with the use of non-steroidal anti-inflammatory drugs, it began to progress rapidly until it became incapacitating, and the patient was taken to the emergency room, where he was evaluated by pain Medicine.

**Figure 1:**
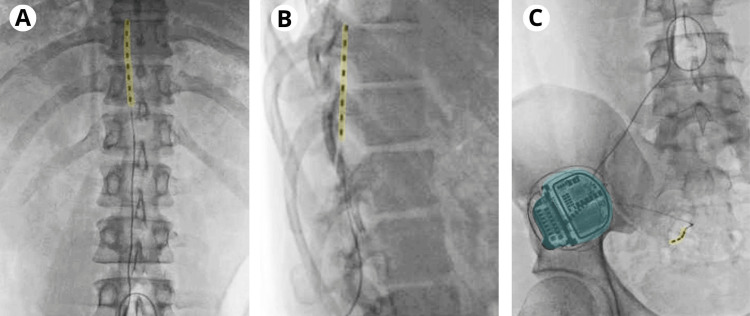
Neurostimulation electrodes locations (A) Anteroposterior view of the electrode (yellow) for spinal cord stimulation tunneled to the right side; (B) lateral view of spinal cord neurostimulator (yellow); (C) fixation of InterStim™ neurostimulator (yellow) and battery (light blue) in the lumbar region.

After a detailed clinical evaluation by a specialist in algology, the diagnosis of intractable pain associated with L2-L3 radiculopathy and post-surgical neuropathy of the genitofemoral nerve was concluded. The severity of the pain and the response to pharmacological treatments based on pregabalin, opioids, and other analgesics were evaluated, concluding that the pain was refractory to medication. Given this, the placement of differential target multiplexed spinal cord stimulation therapy (Medtronic) was decided for treatment. To determine the effectiveness of the proposed approach, a sensory-motor test with electrostimulation was performed in the fluoroscopy room under sedation and with a sterile technique in a prone position with local anesthesia at the incision sites. An electrode guide was placed at L1-L2 level to be able to position the electrode at the epidural space of T8-T9 level and perform the electrostimulation test, which was positive for groin and anterior thigh pain but not for testis. Subsequently, from a lateral approach, the neurostimulator electrode was inserted to be fixed at the S3 level (Figure 1), resolving lower scrotal pain. Therefore, the simultaneous installation of a recharge-free InterStim™ neurostimulator (Medtronic) was considered for the total management of painful areas. The patient came for a check-up 10 days later, reporting adequate improvement in pain, so it was decided to program the placement of a permanent subdermal neurostimulator.

In the fluoroscopy room, with similar conditions to the sensory-motor test, a stimulator generator was surgically placed. The electrodes were fixed at the desired level after tunneling and migrating them in a left-lateralized manner, considering that abdominal stimulation was presented by positioning the electrodes on the right side of the medullary canal in the tests. After carrying out a new stimulation test with the patient awake, the analgesic effectiveness of the electrostimulation was confirmed, and the electrodes were fixed on the lumbar spine to have an analgesic effect at the L1-L2 dermatome levels. Due to clinical improvement and the absence of adverse events, the patient was discharged for follow-up in the outpatient clinic. Subsequent biweekly clinical and technical check-ups, accompanied by a biomedical engineer expert in neurostimulator programming, for three months demonstrated a 90% decrease in pain, as well as reincorporation into his work, domestic, and sexual activities.

## Discussion

Neurostimulation-based interventions have emerged as an option for cases of intractable postsurgical neuropathic pain, such as this case associated with a hernioplasty. These techniques have been improved through technological development, surgical planning, device placement, and postoperative care, so they should be considered for specialized use in pain management due to their high efficacy and safety [8], either in their modality of electrodes that stimulate the anterior portion of the spinal cord, as well as InterStim™ stimulators that interact more directly with a nerve branch.

Spinal cord electrostimulation using the InterStim™ neurostimulator device offers an effective and safe solution for the treatment of painful neuropathies refractory to conservative management of pain syndromes secondary to surgical interventions (such as analgesic-based pharmacotherapy, both with opioids and nonsteroidal anti-inflammatory drugs), like in this neuropathy of the genitofemoral nerve after inguinal hernioplasty. However, the potential for neurological damage to sensory and motor nerves is an inherent risk in any surgical procedure [2], so it must always be performed by qualified professionals in optimal conditions. Similar to what was previously described in the literature [3-5], neurostimulators have shown considerable effectiveness in the management of pain syndromes related to surgical interventions. Since painful neuropathies are complex entities that often include damage at different levels, a single electrode will not necessarily be enough to recover sensory well-being in its entirety, so considering using more than one neurostimulator is viable. However, it is important to seek efficiency by performing as few interventions as possible to obtain the expected clinical outcome in pain management [11]. Finally, this case report not only shows the desired analgesic success but also the functional recovery of the patient to satisfactorily perform his sexual activities and daily life, demonstrating the benefit and justification of using two electrodes in different sites to stimulate, based on the specific needs of the individual. Personalization and patient feedback are essential to obtaining the best clinical outcome.

## Conclusions

Surgical adverse events, such as peripheral nerve injury, are an imminent risk due to the nature of this type of treatment, which generally offers greater gains. However, it is essential to have the largest possible arsenal of minimally invasive interventions in algology and pain medicine in order to offer the best possible relief and recovery of function. Neurostimulation has proven to be a customizable tool according to the individual characteristics of each patient and their clinical presentation, so its mastery by specialized pain centers represents a solution for chronic pain, a global public health problem. Additionally, the reincorporation of his sleep and mental health quality, as well as his previous sexual life, confirms the importance of the negative biopsychosocial impact that an inefficient approach to pain has.

## References

[1] MacPherson R, Pattullo G (2020). Management of postsurgical pain in the community. Aust Prescr.

[2] Laughlin RS, Johnson RL, Burkle CM, Staff NP (2020). Postsurgical neuropathy: a descriptive review. Mayo Clin Proc.

[3] Xu J, Sun Z, Wu J (2021). Peripheral nerve stimulation in pain management: a systematic review. Pain Physician.

[4] Garcia K, Wray JK, Kumar S (2023). Spinal Cord Stimulation. https://pubmed.ncbi.nlm.nih.gov/31985947/.

[5] Tapias Pérez JH (2022). Spinal cord stimulation: beyond pain management. Neurologia (Engl Ed).

[6] Laughlin RS, Rubin DI (2023). Electrodiagnosis: how to read electromyography reports for the nonneurophysiologist. Neurol Clin.

[7] ter Meulen BC, Peters EW, Wijsmuller A, Kropman RF, Mosch A, Tavy DL (2007). Acute scrotal pain from idiopathic ilioinguinal neuropathy: diagnosis and treatment with EMG-guided nerve block. Clin Neurol Neurosurg.

[8] Deer TR, Russo MA, Grider JS (2022). The Neurostimulation Appropriateness Consensus Committee (NACC): recommendations for surgical technique for spinal cord stimulation. Neuromodulation.

[9] Deer TR, Russo M, Grider JS (2022). The Neurostimulation Appropriateness Consensus Committee (NACC): recommendations on best practices for cervical neurostimulation. Neuromodulation.

[10] Deer TR, Pope JE, Lamer TJ (2019). The neuromodulation appropriateness consensus committee on best practices for dorsal root ganglion stimulation. Neuromodulation.

[11] Deer TR, Grider JS, Pope JE (2022). Best practices for minimally invasive lumbar spinal stenosis treatment 2.0 (MIST): consensus guidance from the American Society of pain and Neuroscience (ASPN). J Pain Res.

